# C-low threshold mechanoreceptors: a novel target for health-tech in an increasingly touch-deprived world

**DOI:** 10.1098/rstb.2024.0463

**Published:** 2026-09-17

**Authors:** Francis McGlone

**Affiliations:** Manchester Metropolitan University, Manchester, UK

**Keywords:** affective touch, c-tactile afferents, stress, massage, neurodevelopment

## Abstract

This article describes the putative role of a population of unmyelinated cutaneous nerves that respond optimally to a caressing touch. Massaging touch is of course ‘digital’, dating back to its use by ancient civilizations for its therapeutic properties, where it was found to promote healing, relieve pain, reduce stress and improve overall well-being. What was then non-verifiable ‘alternative medicine’ is now verifiable ‘touch medicine' with the discovery of a population of cutaneous mechanosensitive c-low threshold mechanoreceptors (C-LTMRs) that preferentially encode gentle dynamic touch, projecting centrally to dorsal posterior insular cortex. They are understood to provide a singular vital protective function across the lifespan, from the nurturing touch of the mother to end-of-life palliative care. Early life touch deprivation causes lifelong adverse neurodevelopmental consequences, with recent evidence finding a potential neuroregenerative and neuroprotective role for C-LTMR stimulation in dementia, brain damage, stress resilience and schizophrenia. By analogy with the vital role vitamins play in physical health, affective touch and its neuronal substrate, the C-LTMRs, can be described as ‘vitamin-T’ for neurological health. A case is made here for their therapeutic influence in functional neurological disorders.

This article is part of the discussion meeting issue ‘Digital healthcare for the management of functional neurological disorders’.

## 1. Introduction

A prerequisite for treating any neurological disorder, or any clinical disorder for that matter, is identifying potential causal mechanisms. However, these are not always known and often depend on the limitations of what is currently known, with some of this knowledge often unsupported by empirical evidence, or essentially non-verifiable, e.g. relying on faith. An example of such can be found in some types of nociplastic pain conditions where there is no observable tissue damage or lesions in the pain processing pathways and therefore can be challenging to treat owing to the absence of specific physical findings, coupled with the subjective nature of the symptoms. It differs from nociceptive pain, which is owing to tissue damage, and neuropathic pain, which is caused by damage or dysfunction of the peripheral or central nervous system and helps explain unexplained chronic pain beyond obvious pathology, regardless of its origin from nociceptive, neuropathic, emotional or mixed pain components [1,2]. The quality and body locations of nociplastic pain is also distinct in terms of its intensity and associated central nervous system (CNS) symptoms such as chronic fatigue, disrupted sleep patterns, and mood disturbances such as depression and anxiety. This example of chronic pain states with no discernible cause mirrors a different idiosyncratic group of conditions which are the topic of this *Proceedings B—*‘Digital healthcare for the management of functional neurological disorders’ (FNDs). FNDs (sometimes called conversion disorders) present with a bewildering array of quasi-clinical conditions to the neurologist, without any discernible structural damage or identifiable pathology. Symptoms include seizures, sensorimotor disturbances, disrupted cognitive processes, slurred speech and visual disturbances, weakness and/or paralysis, memory problems, fatigue, etc. The current consensus is that FND is not faked and that sufferers have no conscious control over their symptoms, the heterogeneity of which confounds any simple diagnosis re common mechanisms, leaving the sufferer in danger of being dismissed by clinicians as faking, catastrophizing or imagining their symptoms. A clinical diagnosis of FND can sometimes be based on positive findings, such as Hoover's sign for leg weakness, and often coexists with chronic stress, trauma, pain, or other psychological factors. FND is often ‘accompanied’ by stress, depression and anxiety, but then these could well be effects rather than causes, exacerbating the clinical conditions. It is also found that women are disproportionately affected by FND, with rates of approximately 70% in most large studies [3]. Historically, FND was seen as a hysteria disorder specific to women, and while that framing is no longer accepted scientifically, it is probably that such a prejudice still influences its diagnosis and research focus today [4].

Chronic stress is the cause of many adverse changes in the brain and the body: increased release of glucocorticoids, widespread inflammation, changes in neuronal morphology, dysregulated myelination in prefrontal cortex (PFC) and hippocampus (HC), reduced levels of neurotrophic factors, alterations in brain connectivity, especially within PFC-amygdalo-HPC networks, and circulation disruption [5,6]. It has also been found to be an antecedent of cognitive impairment and dementia.

A growing body of research is showing that affiliative tactile interactions in social mammals, such as allogrooming, have the effect of buffering against neurobiological and behavioural effects of stress [7]. A recent global example of how important affiliative tactile interactions are to well-being can be seen from the COVID pandemic and the reported adverse consequences on people's stress levels and subsequent mental and physical health [8–10]. The adverse consequences of tactile deprivation in solitary confinement have been known for a long time [11].

But what is the mechanism?

Here, a case is made for the role of a population of cutaneous low-threshold thermo-mechanosensitive c-fibres, called c-tactile afferents (CT), as a neurobiological substrate responsible for regulating resilience to stress and therefore potentially a novel digital^ 1^ target for health-tech in an increasingly touch-deprived world, with the potential for management of functional neurological disorders.

## 2. Background to the mechanism

The sense of touch has generally been understood as serving a discriminatory function where tactile stimuli are transmitted by low-threshold mechanoreceptors coupled to fast-conducting myelinated nerves (LTMs) to the primary somatosensory cortex, where a neural map of the body is represented with disproportionate allocations of cortical territory allocated to body parts with higher densities of LTMs, such as the digits and perioral regions. Such a ‘fast’ system provides an evolutionary advantage, allowing the immediate detection of anything touching the body, or being touched by the body, that may require immediate attention [12]. The high conduction velocity of myelinated A-β afferents is a critical feature for discriminative touch, as well as for the guidance of motor activities. However, it has also been recognized for millennia that touch has another more social and affiliative property, playing a vital role in developing intimate relationships and reinforcing social bonds [13]—a recently discovered, in humans, ‘slow’ system of cutaneous nerves. There has been a shift in focus from examining discriminative touch to its less understood social/affiliative role and here a major discovery was made by a Swedish neurophysiologist Ingve Zotterman [14] who, when recording electrophysiological activity from the saphenous nerve of a cat, discovered a C-fibre that responded vigorously to a low force, stroking touch—a C-low threshold mechanoreceptor (C-LTMR)—that he thought, erroneously, might be responsible for the sensation of tickle. C-LTMRs were subsequently found in other mammals, but with no electrophysiological techniques available to record from single fibres in human peripheral nerves, it was presumed that such a type of nerve was lost over evolutionary time. Until that was, the development of an electrophysiological technique by Swedish neuroscientists that enabled recording from single afferent nerves in awake humans—‘microneurography’—that involves inserting a fine tungsten microelectrode, percutaneously, into a peripheral nerve and searching for spike activity from a single nerve fibre within a fascicle in response to stroking across the innervation territory of the nerve. When a unit is found, its response properties can be investigated by mapping its receptive field, force sensitivity, and observing its adaptation rate if a myelinated A-beta afferent, or other properties if an unmyelinated c-afferent. It was using this technique that in 1990, Magnus Nordin [15] first discovered the human analogue of a C-LTMR whilst recording from the supraorbital nerve, where they are called C-Tactile (CT) afferents to distinguish them from non-human animals. It was another Swedish neurophysiologist, Ake Vallbo [16], who played a major role in characterizing their properties, over the next around 30 years.

CTs are uniquely responsive to gentle stroking movements that are commonly perceived as pleasant, have a conduction velocity approximately 50 times slower than myelinated fibres, and are understood to play an essential role in processing salient affective information in response to affiliative tactile behaviours [17,18]. They project primarily to the dorsal posterior insular cortex, as well as to the posterior temporal sulcus, medial prefrontal cortex (mPFC) and dorso-anterior cingulate cortex (dACC) [19]—cortical regions responsible for the integration and output of emotional and social regulation that also have strong connections to the amygdala and hippocampus. A key property of relevance is the influence on neuroendocrine and stress responses [20], an influence that will be expanded on later. Examples of behaviours that produce heightened stimulation of C-LTMR/CT afferents include licking and grooming in rat pups, allogrooming in chimpanzees, maternal nurturing of the neonate, and certain types of massage [21–24]). Such research has now provided a more complex, two-tiered approach to tactile perception with a ‘first’ fast acting A-fibre cutaneous system generating distinct perceptual qualities (*sensing*), followed by a ‘second’ slower acting C-fibre system processing the emotional function of such touch (*feeling*). C-LTMR/CT are now being understood to provide a singular vital protective function, across the lifespan, from nurturing maternal touch to end-of-life palliative care, sharing that protective role with the other two classes of afferent C-fibres, the nociceptors and the pruriceptors. By analogy with the vital role vitamins play in physical health, affective touch and its neuronal substrate the C-LTMR/CT, can be described as ‘Vitamin-T’ for the body and mind. It is this second ‘emotional’ system and the consequences of reduced stimulation of CTs that is here proposed will provide a novel target for digital health-tech in an increasingly touch-deprived world.

## 3. Evidence for an affective touch mechanism

Support for CT afferents being the neurobiological substrate responsible for processing the hedonic properties of affective touch is found in research examining patients with a selective loss of fast-conducting myelinated A-β LTMs. A paper by Olausson *et al.* [25] reported that neuronopathy patients, who lack fast conducting A-β fibres, could still perceive light touch on the forearm, but not on glabrous skin (non-hairy skin) such as the palm, where there are no proven functional CT afferents. Additionally, functional magnetic resonance imaging (fMRI) during CT afferent stimulation showed activation in the dorsal posterior insular cortex and no activation in somatosensory cortices, the normal cortical target area for LTMs. The specificity of CT afferents to slow-moving gentle touch has led to the realisation of the importance of such a niche cutaneous system as captured in the Social Touch Hypothesis [26–28], which proposes that CTs are the neurobiological substrate regulating affective and affiliative behaviours. The skin, and its CT innervation, has been described as a ‘social organ’, that when stimulated at CT-preferred forces, temperatures and velocities regulates stress responses [7,29], lowers blood pressure, releases oxytocin [30] and project to brain regions rich in µ-opioid receptors involved in social attachment and bonding. A lack of nurturing touch, the preferred stimulus for CTs, during infancy contributes to the formation of psychiatric illnesses such as depression, anxiety and cognitive functioning later in life [31–34], as well as de-regulating the hypothalamic-pituitary-adrenal axis (HPA-axis). During infancy, plasticity of developing neural circuitry is at its peak [35,36], with adverse environmental influences at this early stage of life having long-lasting effects into adulthood, with potential adverse repercussions on health. Being an adaptive system, the HPA-axis is responsive to adverse environmental influences such as tactile neglect, which for CTs also involves temperature as they are both velocity and temperature tuned, that can trigger a cascade of events: the release of corticotropin release factor (CRF) from the hypothalamus, which triggers the release adrenocorticotropic hormone (ACTH) from the pituitary gland leading to the release of corticosteroids from the adrenal gland. The amount and frequency of released corticosteroids equate to the intensity of the stress response. This HPA-axis reactivity is modulated by glucocorticoids, regulating CRF synthesis and attenuating its response when the stressor resolves. Evidence for how important a fully functioning stress regulatory system is for health and well-being can be seen with the underdeveloped glucocorticoid systems in the hippocampus and frontal cortex of rat pups that have been deprived of maternal touch and subsequent stress hyperactivity [37]. Maternal licking and grooming of pups are also a trigger for oxytocin (OT) release [38], acting as a natural antidepressant protecting the brain from the adverse effects of stress. OT receptors in limbic regions of the cortex are involved in emotional regulation [39] as well as interacting with dopamine and serotonin to regulate motivational and affective processes. These receptors release OT when CTs are stimulated, inhibiting the stress response by dampening amygdala activation during stressful situations [40] and promoting affiliative behaviours, evidencing OT's role in forming attachments [41,42]. CT stimulation leads to increased release of 5-HT in the central amygdala (CeA), commonly associated with elevated mood states, and is also important in forming social bonds and social perceptions [43]. Neglect and tactile isolation have profound effects on the binding and synthesis of 5-HT with repeated isolation of rats decreasing serotonin binding in the ventral hippocampus, leading to anxiety-like behaviours—the ventral hippocampus modulates anxiety-like behaviour in rats with serotonergic transmission within the hippocampus facilitating adaptation to stress. This finding is mirrored in children reared in neglectful families who were found to be at a higher risk of delinquency later in life, and had lower levels of serotonin [44]. Further evidence of a putative link between CT stimulation, 5-HT and social touch is the psychostimulant drug 3,4-methylenedioxymethamphetamine (MDMA), an indirect monoaminergic agonist stimulating the release and inhibiting the reuptake of serotonin leading to feelings of empathy coupled with a heightened need and reward from physical closeness with others [45,46]. MDMA increases social behaviour in animal models and has shown promise in psychiatric disorders, such as autism spectrum condition (ASC) and post-traumatic stress disorder (PTSD). Abnormal levels of both OT and 5-HT have been reported in ASC, a condition where gentle, slow stroking touch can lead to negative reactions to what is typically considered a pleasant stimulus ([19,47]; see also [48])). These results are indicative of serotonin's essential role in developing social behaviours, reinforcing the necessity to provide skin-to-skin touch during infancy to adequately stimulate CT afferents and prevent adverse neurodevelopmental consequences later in life.

Of interest here is the potential role of neurodevelopmental sequelae in the recognised comorbidities between ASC and FNDs, such as emotion regulation and recognition, sensory hyper/hypo sensitivity, proprioception and interoception [49]. Comorbidities can arise from shared genetic pathways (e.g. serotonin, dopamine) and environmental stressors (e.g. neglect, trauma) adding to the complexity of diagnosis and treatment. Findings such as these reinforce the impact of early life stress on synaptic circuitry, particularly in brain regions responsible for social and emotional cognition, providing further evidence that childhood tactile neglect can hinder the development of cortical regions involved in the HPA-axis response. Infants from a family where there is minimal skin-to-skin touch and are in an environment where the infant is ignored, are more likely to develop a dysfunctional stress response and the health consequences associated with higher allostatic load. Prolonged activation of the HPA-axis increases allostatic load with the adverse effects the stress response has on the body owing to repeated neuroendocrine activation, resulting in mental and physical health deterioration [50]. McEwen & Wingfield [51] and Taylor *et al.* [52] further supported this point by finding that a neglectful and highly stressful environment increases the vulnerability to later physical and mental health deterioration, as well as higher and prolonged cortisol production [53]. Bowlby's Attachment Theory [54] highlights the importance of touch during infancy in regulating the stress response, suggesting that children learn coping skills through secure attachments, providing a secure base of emotional support to regulate stress reactivity. Secure attachments are built on skin-to-skin contact, coupled with affective behaviours between mother and infant [55]. Children receiving less affective touch are at risk of forming insecure or avoidant attachments, leading to developing inappropriate coping skills and the risk of HPA-axis hyperactivity owing to an inability to cope with life's stressors effectively. Furthermore, institutionalization studies have found that early adversity during childhood can interfere with white matter development in brain regions responsible for social interactions, and later difficulties in regulating emotions (see review by [56]). Early deprivation in institutionalized Romanian orphans also showed neurocognitive impairment, impulsivity and social deficits [57], as well as intellectual, physical, behavioural and socio-emotional deficits [58]. Therefore, neglect, early life stress and minimal skin-to-skin contact contribute to adverse neurological changes. These critical studies have previously been explained by behavioural models of attachment, such as the infant simply seeking the mother to feel safe and secure. However, the social touch hypothesis and the affective role of CT afferents can now explain this phenomenon in more depth; Harlow's monkey study and Bowlby's attachment theory demonstrate the infant's innate desire to seek CT afferent stimulation in an attempt to feel safe and secure (protected), thus dampening stress reactivity. Neglecting these needs may manifest as neurodevelopmental and behavioural disturbances owing to understimulated CTs. Additionally, the social touch hypothesis appreciates maternal touch as a necessity and not merely as an indulgence [59]. One can infer from this that positive touch experiences in early life would help buffer against adverse childhood experiences, mitigating the risk of FND.

However, touch is also crucial for the developing foetus, which responds to gentle stroking movements of fine lanugo hairs in utero at around 8.5 weeks [60], a response occurring before myelinated LTMs have developed [61]. Bystrova *et al.* [62] proposed a mechanism that facilitates the development of the foetus via repeated oscillations of fine lanugo hairs, with the function of activating brain regions such as the hypothalamus and insular cortex, therefore suggesting that activation of CT afferents primes the social brain as early as 8.5 weeks into gestation. Embryologically, not only are sense organs developed from the skin, but the spinal cord and brain are infolded and developed from ectodermal (skin) cells. This embryological skin–brain connection has a profound consequence for the emerging ‘sense of self’ during development, with the discovery of CTs as the potential afferent candidate providing the initial scaffold of the embodied brain. Synapse growth is at its maximum levels during the first year of birth, partaking in cell maturation (dendrite and axon growth) and synaptogenesis [36]. However, when pre-term babies are placed in an incubator in neonatal intensive care units (NICU), this synaptic development can be severely hindered since there is no uterine or maternal touch (the foetus responds to touch of the mother's abdomen [63]) to excite CT afferent pathways to stimulate synaptic growth. Babies born pre-term have been shown to be at higher risk of developing autistic-like traits, psychiatric illness and increased prevalence of a variety of physical health conditions [31,64,65]. Research examining the effects of kangaroo mother care on the development of pre-term babies supports the necessity of stimulating CTs through maternal skin-to-skin contact and their vital role in improving health outcomes and cortical development, as well as stress regulation and attachment formation [66]. Weiss *et al.* [67] observed mothers’ behaviour with their pre-term baby and found that the babies of mothers who used more stimulating tactile behaviours had improved visual motor skills at one year of age, demonstrating that gentle touch could compensate for neurosensory deficits commonly observed in pre-term infants.

The functional role of CTs, although initially described as subserving a hedonic function, is now better described as a key input regulating stress and thereby providing a fundamental protective role, a process that starts *in utero* and continues across the lifespan. While stimulation of CTs does not directly regulate the HPA-axis it can modulate stress responses via the following pathways:

— CTs project to the posterior insula, then the anterior insula, anterior cingulate cortex (ACC) and orbitofrontal cortex—areas involved in emotional regulation.
— CT-mediated touch is linked with increased oxytocin release, buffering stress and reducing HPA-axis activation.
— CT touch increases vagal tone and heart rate variability, promoting parasympathetic dominance, reducing cortisol release and regulating allostatic load.
— Repeated exposure to CT-optimal touch—such as parental caressing or affectionate touch in relationships—can reduce chronic stress burden, thus reducing allostatic load over time.

Although regulation of the HPA axis and allostatic load was not originally seen as the primary functional role of CT afferents, with the Social Touch Hypothesis [28] seeing them as more directly involved in processing affective aspects of touch, the emerging view is that CTs contribute to stress buffering and emotional homeostasis through their engagement with social, emotional and neuroendocrine systems. It is on this basis that an opportunity is here proposed where CT stimulation can provide a novel digital^2^ target for health-tech in an increasingly touch-deprived world, with the potential for management of functional neurological disorders.

## 4. A ‘digital’ solution

The preceding section has made a case for a stress regulatory role from the stimulation of CTs, as is the case using the hands during massage (digital!), which, as has been describes above, downregulates the HPA-axis (lowering cortisol levels, etc.) and thereby ameliorating the adverse effects on mental and physical health of reduced CT stimulation—a modern ‘scourge’ owing to the attention-grabbing and addictive lure of digital platforms such as Tik Tok, Facebook and Instagram. Empirical support for the role of affective touch in stress resilience has been shown recently in rodent and human models. In one study, male rats were subjected to an unpredictable chronic mild stress (CMS) procedure over a two-week period where one group was stroked daily at C-LTMR optimal velocity (5 cm s^−1^) and another group at non-C-LTMR velocity (30 cm s^−1^)—for just 10 min d^−1^. A third CMS- exposed group did not receive any tactile stimulation. At the end of the two weeks, serum corticosterone levels, anxiety and depressive-like behaviours in these three groups were assessed, in comparison to a control group of non-CMS-exposed rats. C-LTMR velocity stroking significantly reduced CMS-induced elevations in corticosterone following an acute forced swim, with significantly attenuated anxiety-like behaviours (e.g. elevated plus-maze) than the other CMS-exposed rats. These findings support the theory that C-LTMRs play a functional role in building resilience to stress by regulating physiological and behavioural states [7]. For the human model, two populations of children (age 4–10) were recruited: (i) a cohort living in an orphanage and (ii) a fostered cohort, with both groups receiving CT-directed touch daily for just 10–15 min, for 5–6 weeks. An age-matched cohort of children living in a family environment acted as a control group and did not receive any scripted tactile stimulation. Salivary cortisol was collected at the beginning (T1) and at the end (T2) of the study in all three groups. For institutionalized and fostered children there was a significant improvement in the level of cortisol (*p <* 0.0001) between T1 and T2, which is manifested in the balancing cortisol levels: a decrease where it was elevated and an increase where the critically low level testified to the distress of the child. Balancing cortisol levels is a process of recovery to normal values, which indicates the restoration of neurohumoral mechanisms of stress regulation in response to daily CT-directed touch. The effect of balancing cortisol levels was more pronounced in the group of fostered children compared to the group of orphanage children (*p* = 0.03) [24]. This study was carried out in Russia, where fostering has been reported as being a remunerative enterprise and not an altruistic one, but by all means not generally the case (see [68]). The children in the control group had no significant differences. What these two examples tell us is that ‘digital touch’ targeted to stimulate CT receptors produces phasic and tonic nervous system changes that enhance affect, reduce stress and facilitate social processes with potential long-term benefits via epigenetic mechanisms and learning [24].

Further support for the influence of C-LTMR targeted stimulation on neurological disorders can be found in research on dementia and schizophrenia. In a dementia study the effect of neonatal tactile stimulation, delivered by gently stroking the head/back of the mouse, (15 min 3× day-^1^ over 15 days) on neurological symptoms in an Alzheimer disease mouse model found that such stimulation improved cognition, motor skills and anxiety-like symptoms in both gestationally stressed and non-stressed adult transgenic mice genetically modified to express the human amyloid precursor protein (APP) gene. The study reported reduced A-β plaque formation [69]—the only intervention being relatively brief daily periods of C-LTMR directed touch (see also [70]). In an animal model of schizophrenia, Sampedro-Viana *et al.* [71] found that neonatal handling (NH)—gently stroking rats twice a day for 3–4 minutes—increased social interaction preference and that the increase in social behaviour/preference correlated with differences in c-Fos expression levels in brain areas that integrate the ‘social brain’.

By analogy with the pharmacokinetic profile of a drug and its half-life, a key observation from these empirical studies is the ‘tactokinetic’ profile of touch interventions as the beneficial effects were all seen with relatively small ‘doses’, i.e. a matter of minutes per day of C-LTMR/CT directed touch.

## 5. Conclusion—implications for health care

CT stimulation has a number of benefits (see table 1). Benefits of C-tactile (CT) stimulation treatments using affective touch such as gentle massage, body-oriented therapy or somatic mindfulness, might help reintegrate sensory–emotional signals and restore a coherent sense of body ownership. Research increasingly suggests that affective and interoceptive processing abnormalities may contribute to FND symptoms, such as altered integration of sensory and emotional signals, with insula and limbic regions misinterpreting certain bodily signals. This could lead to a 'disconnect' between how the body feels (affective/interoceptive sense) and how it is represented consciously. Regarding reported diminished affective touch sensitivity, studies show that some FND patients rate affective touch (slow, gentle stroking) as less pleasant than controls, even though they can detect it, suggesting an altered interpretation or emotional processing of tactile input [72,73]. Such an effect may be owing to abnormal predictive coding in FND where prediction errors may be resolved incorrectly, either by changing perception (feeling numbness) or action (involuntary movements). One putative role of CTs is in ‘anchoring’ the sense of body ownership, so its disruption could exacerbate FND symptoms.

**Table 1.** Benefits of C-tactile stimulation treatments.

benefit	description
reduces stress and anxiety	lowers cortisol, activates parasympathetic system
enhances emotional regulation	promotes feelings of safety and emotional stability
improves social bonding	increases oxytocin and strengthens attachment and close relationships
promotes positive affect	enhances mood, well-being
supports interoception (body awareness)	increases awareness of internal bodily states
alleviates pain	modifies pain perception and reduces its affective dimension
improves sleep quality	promotes relaxation and reduces physiological arousal before sleep
enhances therapeutic outcomes	Improves outcomes in therapies for trauma, depression, autism and dementia when integrated into touch-based interventions

The potential avenues to explore now are: (i) running clinical trials with FND patients using CT-directed touch analogous to a ‘vitamin-T’ drug trial and (ii) exploring the opportunities for making the manually applied ‘digital’ stimulation of CTs a health-tech ‘digital’ solution via CT-stimulating medical devices [74].

## Data Availability

This article has no additional data.

## Footnotes

^1^
Here the word ‘digital’ refers to the use of the hands/fingers to deliver CT-directed touch.

^2^
Here the word ‘digital’ refers to the use of the hands/fingers to deliver CT-directed touch.

## Theme

One contribution of 12 to a discussion meeting issue ‘Digital healthcare for the management of functional neurological disorders’.
